# Development and validation of immune dysfunction score to predict 28-day mortality of sepsis patients

**DOI:** 10.1371/journal.pone.0187088

**Published:** 2017-10-26

**Authors:** Wen-Feng Fang, Ivor S. Douglas, Yu-Mu Chen, Chiung-Yu Lin, Hsu-Ching Kao, Ying-Tang Fang, Chi-Han Huang, Ya-Ting Chang, Kuo-Tung Huang, Yi-His Wang, Chin-Chou Wang, Meng-Chih Lin

**Affiliations:** 1 Division of Pulmonary and Critical Care Medicine, Department of Internal Medicine, Kaohsiung Chang Gung Memorial Hospital, Chang Gung University College of Medicine, Niaosung, Kaohsiung, Taiwan; 2 Department of Respiratory Therapy, Kaohsiung Chang Gung Memorial Hospital, Chang Gung University College of Medicine, Kaohsiung, Taiwan; 3 Department of Respiratory Care, Chang Gung University of Science and Technology, Chiayi, Taiwan; 4 Division of Pulmonary Sciences and Critical Care Medicine, University of Colorado, Denver and Denver Health Medical Center, Denver, Colorado, United States of America; University of Florida, UNITED STATES

## Abstract

**Background:**

Sepsis-induced immune dysfunction ranging from cytokines storm to immunoparalysis impacts outcomes. Monitoring immune dysfunction enables better risk stratification and mortality prediction and is mandatory before widely application of immunoadjuvant therapies. We aimed to develop and validate a scoring system according to patients’ immune dysfunction status for 28-day mortality prediction.

**Methods:**

A prospective observational study from a cohort of adult sepsis patients admitted to ICU between August 2013 and June 2016 at Kaohsiung Chang Gung Memorial Hospital in Taiwan. We evaluated immune dysfunction status through measurement of baseline plasma Cytokine levels, Monocyte human leukocyte-DR expression by flow cytometry, and stimulated immune response using post LPS stimulated cytokine elevation ratio. An immune dysfunction score was created for 28-day mortality prediction and was validated.

**Results:**

A total of 151 patients were enrolled. Data of the first consecutive 106 septic patients comprised the training cohort, and of other 45 patients comprised the validation cohort. Among the 106 patients, 21 died and 85 were still alive on day 28 after ICU admission. (mortality rate, 19.8%). Independent predictive factors revealed via multivariate logistic regression analysis included segmented neutrophil-to-monocyte ratio, granulocyte-colony stimulating factor, interleukin-10, and monocyte human leukocyte antigen-antigen D–related levels, all of which were selected to construct the score, which predicted 28-day mortality with area under the curve of 0.853 and 0.789 in the training and validation cohorts, respectively.

**Conclusions:**

The immune dysfunction scoring system developed here included plasma granulocyte-colony stimulating factor level, interleukin-10 level, serum segmented neutrophil-to-monocyte ratio, and monocyte human leukocyte antigen-antigen D–related expression appears valid and reproducible for predicting 28-day mortality.

## Introduction

In intensive care units (ICUs), infected patients had more than twice mortality rate that of noninfected patients [1]. Infection may cause local organ involvement without generating a dysregulated systemic host response [2]. Sepsis is defined as the presence of an infection together with systemic manifestations [3]. Sepsis complicated by organ dysfunction is termed severe sepsis. Previous study revealed 750,000 patients suffered from severe sepsis annually in the United States (0.3% of total population and 2.26% of hospital discharges)[4] Severe sepsis patients had ICU mortality rate ranged from 21.0–36.7%, and in hospital mortality ranged from 22.0–44.5% based on different patient population and definition of severe sepsis [4–8]. Sepsis, which can be considered a battle between pathogens and a host’s immune system [9], is a life-threatening organ dysfunction due to a dysregulated host response to infection [10]. Immune cells play a critical role in the host’s response to sepsis.

Previous study revealed some patients dying of sepsis have marked immunosuppression [11, 12]. In septic patients, immunological variables behave in a mixed and time-dependent manner [13].

There are several existing scoring systems developed for mortality prediction, such as APACHE II, Sequential Organ Failure Assessment score (SOFA score), and Charlson Index. However, none of these takk immune dysfunction status into account. With a substantial degree of heterogeneity in the sepsis response ranging from cytokines storm to immunoparalysis, better patient stratification is needed [14]. Here we aimed to develop and validate a scoring system that can determine patients’ immune dysfunction status related to outcomes.

## Materials and methods

### Patient enrollment

This prospective observational study was conducted at Kaohsiung Chang Gung Memorial Hospital, a 2,700-bed tertiary teaching hospital in southern Taiwan. The study evaluated patients with severe sepsis or septic shock who were admitted to medical ICUs between August 2013 and June 2016. All patients admitted to participating units were screened for eligibility. Patients were enrolled if they agreed to undergo blood sampling during ICU hospitalization. Patients were excluded if they met either of the following criteria: (1) <18 years of age; or (2) Those who had ICU waiting time longer than 24 hours after diagnosis of sepsis. 3) Those who received granulocyte-colony stimulating factor (G-CSF) 1 week prior to ICU admission. All patients received blood sampling at day 1 of ICU admission. The primary outcome was 28-day mortality (day 1 defined as ICU admission day). All patients were followed until discharge from or death in the hospital. Data of the first consecutive 106 septic patients comprised the training cohort, and of other 45 patients comprised the validation cohort.

This study’s design was approved by the institutional review board of Chang Gung Memorial Hospital, and written informed consent was obtained from each patient or a suitable family member.

### Definitions

The definition of severe sepsis was first adapted from the 2001 International Sepsis Definitions Conference and the Surviving Sepsis Campaign [15]. All enrolled patients fulfilled the definition of sepsis from Third International Consensus Definitions for Sepsis and Septic Shock (Sepsis-3) [16]. We than adapted the new definition. Day 1 was the day of arrival to the ICU.

### Plasma and PBMC preparation

Whole blood samples were drawn from all patients on day 1 of ICU admission and collected in heparinized tube (BD, Franklin Lakes, NJ, USA). All patients received blood sampling on day 1 of ICU admission. Using a Ficoll-Paque (Amersham Biosciences, Uppsala, Sweden), whole blood was centrifuged at 400 *g* × 30 min to separate the plasma and PBMCs. All of the PBMC samples were treated immediately and the plasma samples were stored at -80°C until use. Fresh PBMCs were aliquoted into two parts: one that was used for the monocyte human leukocyte antigen D related (HLA-DR) expression measurement and another that was used for the cell culture.

### Monocyte HLA-DR expression measurement by flow cytometry

HLA-DR–related monocyte expression was measured by flow cytometry (Cytomics FC500; Beckman Coulter, Inc., Fullerton, CA, USA). Staining and cell acquisition for flow cytometry were performed within 1 hour after the blood sampling. Monoclonal antibodies were used as follows: CD14-PerCP/Cy5.5 (clone: HCD14; Biolegend, San Diego, CA, USA), HLA-DR-FITC (clone: L243; Biolegend) per 100 μL of PBMC blood. Negative controls were mouse monoclonal antibodies IgG1, PerCP/Cy5.5 (clone: MOPC-21), IgG2a, FITC (clone: MOPC-173), and IgG2a, PE (clone: MOPC-173), all of which were isotype-matched as recommended by the manufacturer. Monocytes were characterized based on their CD14 expression. At least 30,000 PBMCs were analyzed from each sample. The results are expressed as percentages of HLA-DR–positive monocytes of the total monocyte population.

### Cell culture

PBMCs (1 × 10^6^) were plated in a 5-mL round-bottom polystyrene test tube (BD Falcon, Bedford, MA USA) with 2 mL of sterile DMEM culture medium (Gibco, Grand Island, NY, USA) containing 1% heat-inactivated fetal bovine serum (Gibco), 1 mM L-glutamine, and 1 mM sodium pyruvate. Inflammation was induced using LPS 100 ng/mL (Sigma, St. Louis, MO, USA) or not. The tubes were incubated at 37°C in 5% CO_2_ for 4 hours. Samples of the conditioned media were analyzed for cytokine expression levels.

### Milliplex assay

Cytokine levels of plasma and conditioned media including G-CSF, interleukin (IL)-10, IL-6, and tumor necrosis factor-α (TNF-α) were quantified using a Human Cytokine/Chemokine Magnetic Bead Panel customized Milliplex MAP kit (#HCYTOMAG-60K, EMD Millipore, Darmstadt, Germany). The assay was performed according to the manufacturer’s instructions. Standards and samples were analyzed on a MAGPIX System device (Millipore) by MILLIPLEX® Analyst 5.1 software using a five-parameter logistic curve fitting model.

### Statistical analyses

Statistical analyses were performed using MedCalc (version 14.10.2). Categorical variables were compared using the chi-square test or Fisher’s exact test where appropriate, while continuous variables were analyzed using Student’s t-test or the Mann–Whitney U test where appropriate. A receiver operating characteristic (ROC) curve and Youden's index was used to determine the best cut-off values of the prognostic factors.

Multivariate analyses for independent prognostic factors selection were performed using backward elimination of logistic regression analysis.

These independent prognostic factors were included in the scoring system. Regression coefficient was used to weight the score of each factor. The Kaplan-Meier method and the log-rank test were used to determine the effect of the immune dysfunction scores on patient survival. Kruskal-Wallis test was used for assessing the association between post LPS stimulation immune response and immune dysfunction score. P values <0.05 were considered statistically significant.

### Immune dysfunction score construction

The risk factors identified by multivariate analysis weighted points proportional to the β regression coefficient values were summed to calculate a risk score for each patient.

Patients were re-grouped into high, medium, and low immune dysfunction levels based on different value of immune dysfunction score.

## Results

### Patient characteristics

**Fig 1:**
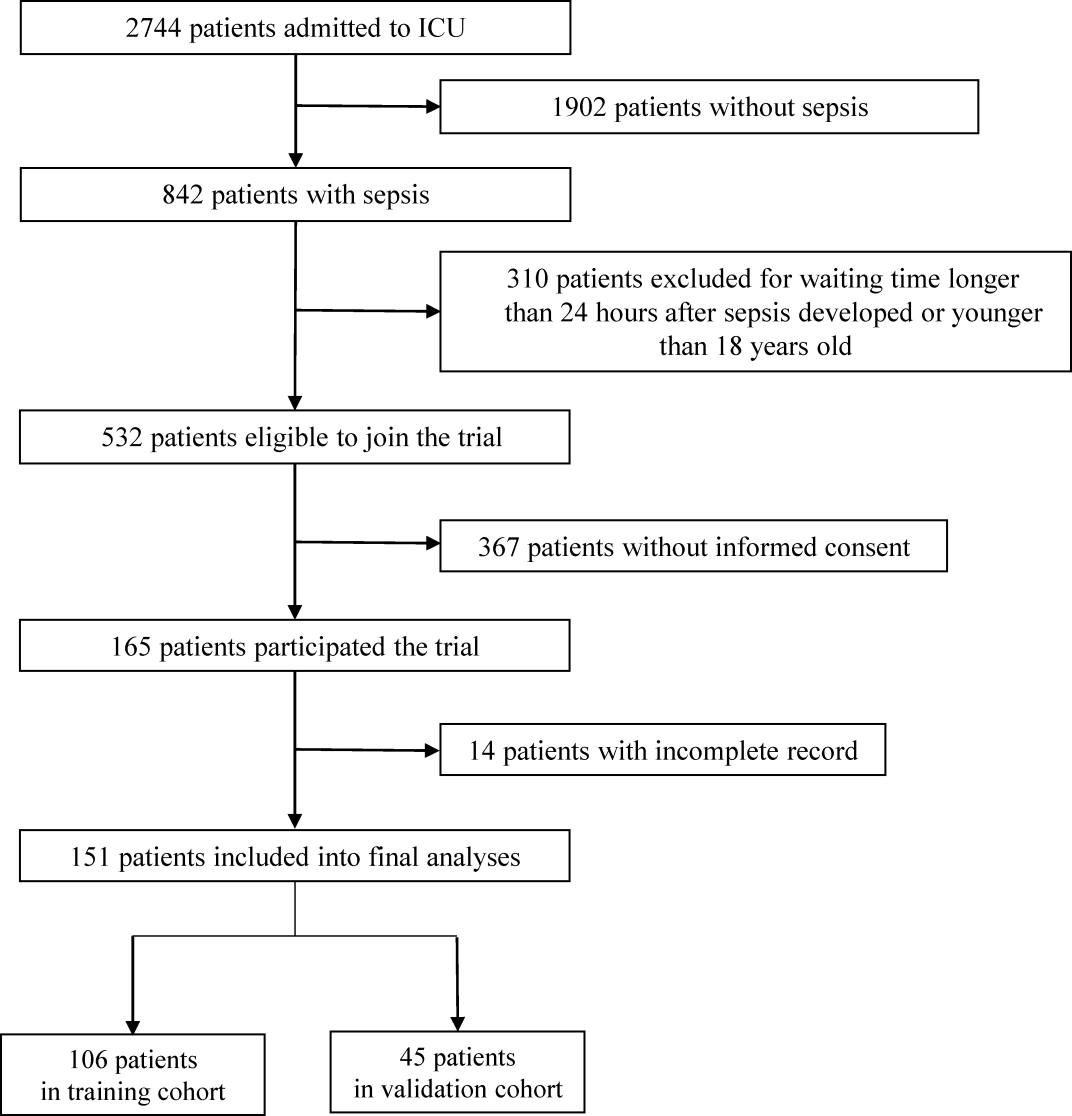
Patient recruitment and assignment.

A total of 2744 patients were admitted into ICUs between August 2013 and June 2016. A total of 824 patients met the criteria for diagnosis with sepsis [16]; of them, 151 were enrolled (Fig 1). The first consecutive 106 septic patients were included in the training cohort to analyze the model construction, while the other 45 patients were assigned to the validation cohort. Among the two populations, 21 of 106 (19.8%) patients in the training cohort and 9 of 45 patients (20%) in validation cohort died within the first 28 days of ICU admission. The patients’ baseline characteristics are presented in Table 1. There were comparable severity levels and co-morbidity rates between survival and non-survival groups. However, there were significant intergroup differences regarding SOFA score as well as segmented neutrophil-to-monocyte ratio (SeMo ratio) (Table 1). Demographics and clinical characteristics between the training and validation cohorts are presented in S1 Table.

**Table 1:** Baseline clinical hematological and biochemical parameters, parameters, SOFA score, and immune profiles of the study patients.

	All (N = 106)	Non-survivor (n = 21)	Survivor (n = 85)	P value
**Clinical parameters**				
Age (years), mean (SD)^a^	68.22 (15.7)	68.19 (14.5)	68.23 (16.2)	0.834
APACHE II score, mean (SD)	26.78 (8.5)	28.55 (8.3)	26.36 (8.6)	0.269
Male, n (%)^b^	61 (57)	11 (52)	50 (58)	0.593
Body mass index, mean (SD)	23.24 (5.1)	24.75 (4.7)	22.86 (5.5)	0.078
Charlson index, mean (SD)	2.42 (1.5)	2.7 (1.5)	2.35 (1.6)	0.104
Cardiovascular disease, n (%)	32 (30)	6 (29)	26 (31)	0.857
Hypertension, n (%)	64 (60)	15 (71)	49 (58)	0.248
COPD, n (%)	18 (17)	2 (10)	16 (19)	0.309
Asthma, n (%)	5 (5)	1 (5)	4 (5)	0.991
Pulmonary tuberculosis, n (%)	4 (4)	1 (5)	3 (4)	0.791
Cancer, n (%)	15 (14)	3 (14)	12 (14)	0.984
Diabetes mellitus, n (%)	55 (52)	13 (62)	42 (49)	0.305
Stroke, n (%)	24 (23)	5 (24)	19 (22)	0.886
Chronic kidney disease, n (%)	28 (26)	8 (38)	20 (24)	0.175
**Baseline SOFS score, hematological and serum biochemical analyses**				
SOFA score, mean (SD)	9.45 (3.6)	12.09 (4.4)	8.8 (3.15)	0.001
WBC, 1000/μL, mean (SD)	16.27 (8.5)	18.61 (10.3)	15.70 (7.9)	0.290
SeMo ratio, mean (SD)	30.97 (50.3)	23.75 (28.30)	32.69 (54.2)	0.046
C-reactive protein, mg/L, mean (SD)	171.79 (128.0)	220.02 (152.0)	160.61 (120.0)	0.208
Procalcitonin, ng/mL, mean (SD)	23.93 (49.7)	29.81 (49.0)	22.43 (50.0)	0.487
**Immune profiles**				
G-CSF pg/mL, mean (SD)	63.09 (104.8)	94.74 (232.5)	53.09 (99.13)	0.033
IL-10, pg/mL, mean (SD)	15.25 (58.05)	82.78 (147.26)	10.62 (31.6)	0.010
IL-6, pg/mL, mean (SD)	43.31 (84.3)	91.16 (473.29)	33.82 (80.8)	0.004
TNF-α pg/mL, mean (SD)	31.15 (36.5)	39.13 (87.8)	28.46 (34.8)	0.048
HLA-DR expression %, mean (SD)	90.30 (18.4)	87.1 (39.9)	92.05 (11.1)	0.030

^a^ continuous variables were analyzed using Mann–Whitney U test.

^b^ Categorical variables were compared using the chi-square test or Fisher’s exact test where appropriate.

Abbreviations: COPD, Chronic obstructive pulmonary disease; G-CSF, granulocyte-colony stimulating factor; HLA-DR, human leukocyte antigen D–related; IL, interleukin; SeMo, segmented neutrophil-to-monocyte; SOFA, Sequential Organ Failure Assessment score.

### Immune status and plasma cytokine expression

Among 106 patients in the training cohort, 2 patients had missing data about SeMo ratio, one had no baseline monocyte HLA-DR expression level. In non-survival patients, there were decreased median baseline HLA-DR monocyte expressions measured by flow cytometry compared to survival patients. Meanwhile, there were significant intergroup differences in G-CSF, IL-10, IL-6, and TNF-α expressions (Table 1).

### Immune dysfunction score

**Fig 2:**
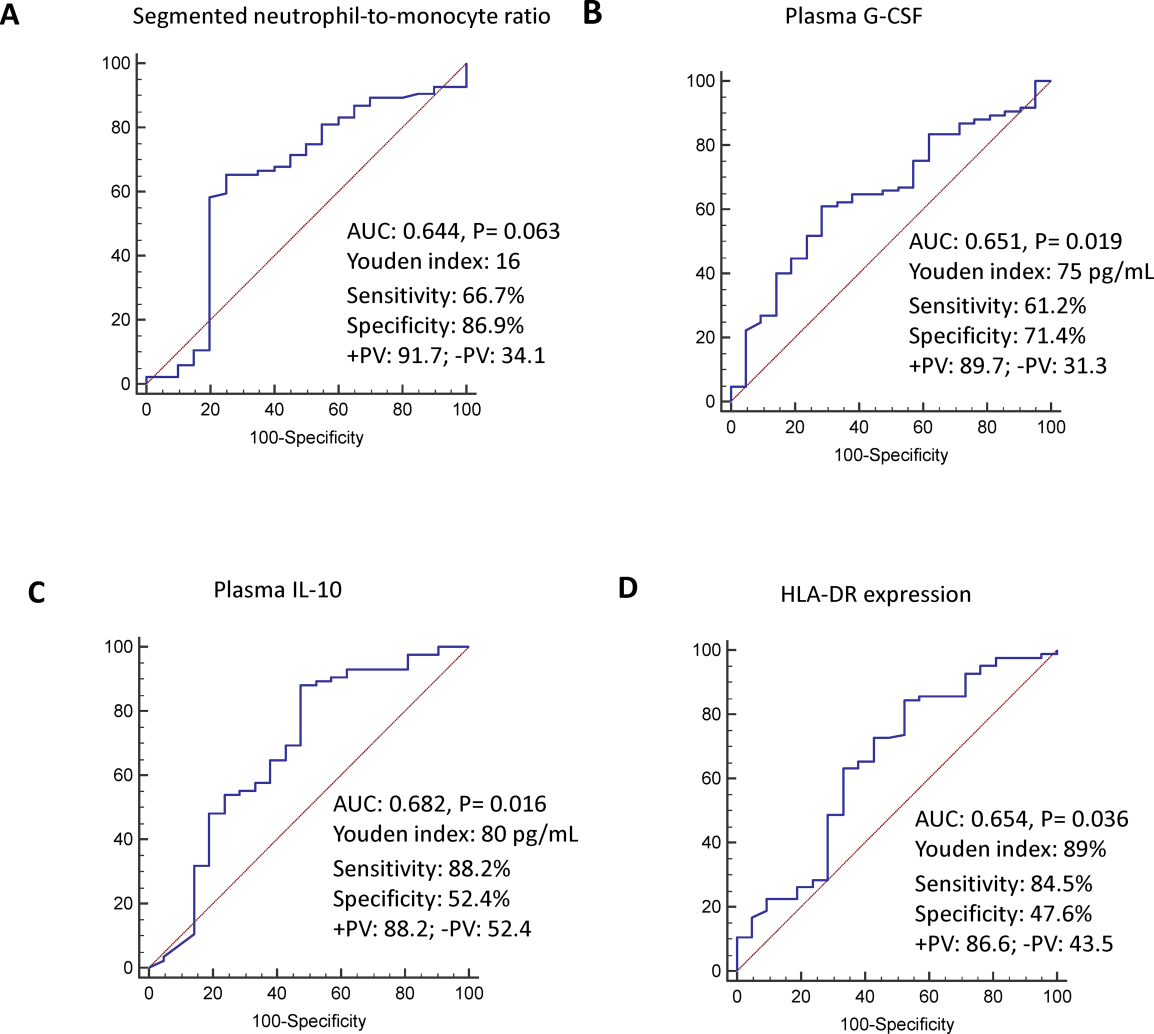
Receiver operating characteristic curve for discriminating between 28-day survivors and non-survivors with sepsis in the intensive care unit using plasma segmented neutrophil-to-monocyte ratio (A), granulocyte-colony stimulating factor level (B), Interleukin-10 level (C), and monocyte human leukocyte antigen-antigen D–related expression (D). The sensitivity and specificity were determined at best cutoffs determined by Youden index.

A ROC curve was used to examine the clinical and immune parameters that were statistically significant on univariate analysis to determine the best cut-off point for 28-day mortality prediction. The best cut-off points for SOFA score, SeMo ratio, and G-CSF, IL-10, IL-6, TNF-α, and monocyte HLA-DR expression were 10, 16, 75 pg/mL, 80 pg/mL, 41 pg/mL, 36 pg/mL, and 89%, respectively. Independent predictive factors identified on multivariate logistic regression analysis included SeMo ratio, G-CSF, IL-10, and monocyte HLA-DR expression (Table 2). The ROC curves for SeMo ratio, G-CSF level, IL-10 level, and monocyte HLA-DR expression had areas under the curve (AUC) of 0.644 (95%CI: 0.544–0.736), 0.651 (95%CI: 0.552–0.741), 0.682 (95%CI: 0.584–0.769), and 0.654 (95%CI: 0.555–0.744), respectively (Fig 2).

**Table 2:** Multivariate analyses for immune dysfunction score parameter selection^a^.

	Regression coefficient	P value	Risk score
SeMo ratio		0.004	
>16	Reference		0
≤16	1.337		1
G-CSF plasma level, pg/mL		0.007	
>75	1.304		1
≤75	Reference		0
IL-10 plasma level, pg/mL		<0.001	
>80	1.853		2
≤80	Reference		0
HLA-DR expression %		0.029	
>89	Reference		0
≤89	1.248		1

^a^Multivariate analyses for independent prognostic factors selection were performed using backward elimination of logistic regression analysis.

Abbreviations: G-CSF, granulocyte-colony stimulating factor; IL, interleukin; HLA-DR, human leukocyte antigen D–related; SeMo, segmented neutrophil-to-monocyte

**Fig 3:**
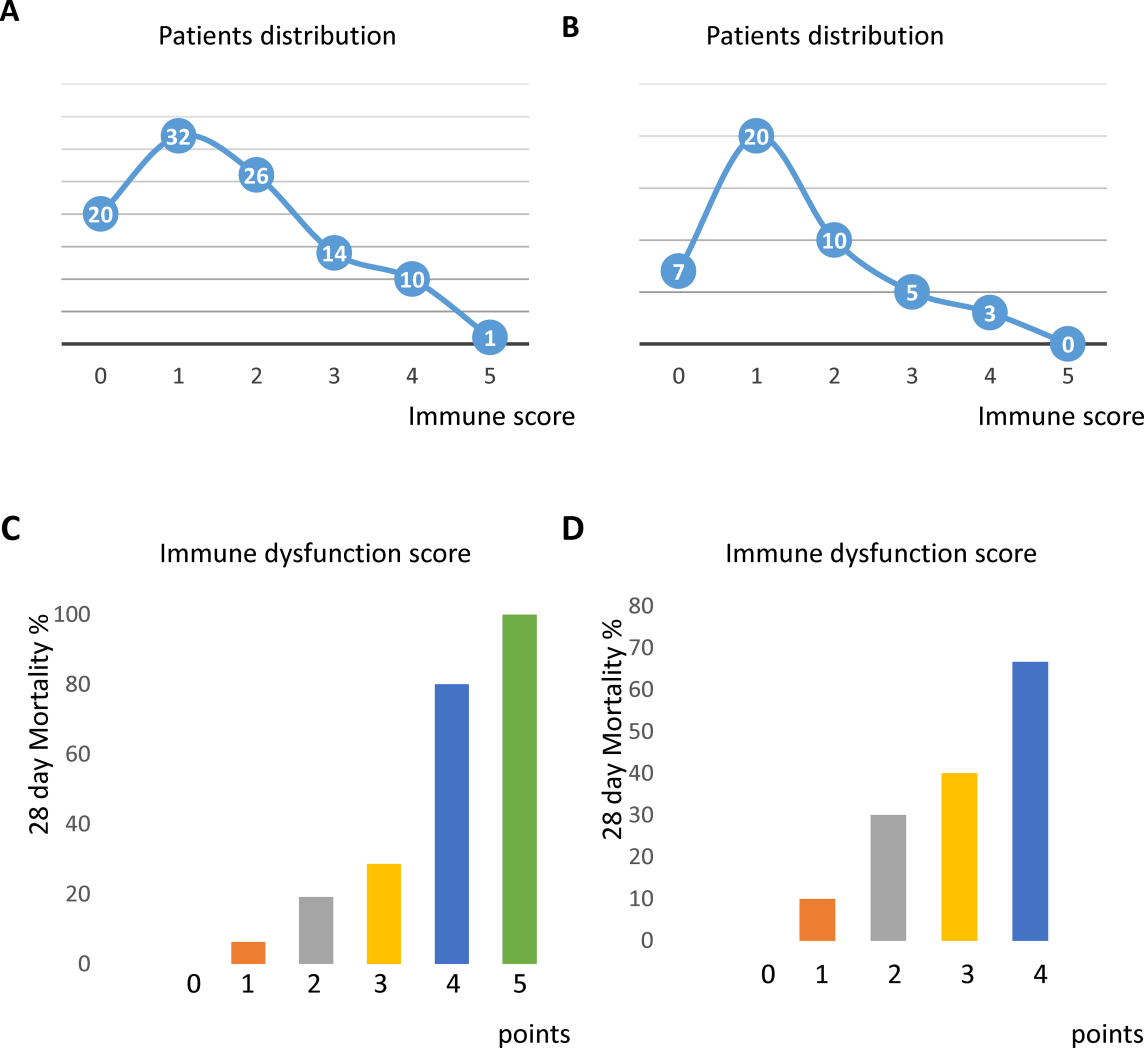
Patient distributions in the training (n = 103) (A) and validation (n = 45) (B) cohort. Immune dysfunction score and 28-day mortality rate in the training (C) and validation (D) cohort.

These independent risk factors associated with 28-day mortality were selected to construct the score in 103 patients in training cohort (Table 2). The patient distributions of immune dysfunction score of the 103 patients in the training cohort (Two patients had missing data about SeMo ratio, one had no baseline monocyte HLA-DR expression level) and 45 patients in the validation cohort are shown in Fig 3A and 3B, respectively. The 28-day mortality rates for patients at the dysfunction scores are shown in Fig 3C. (training cohort) and Fig 3D. (validation cohort).

**Fig 4:**
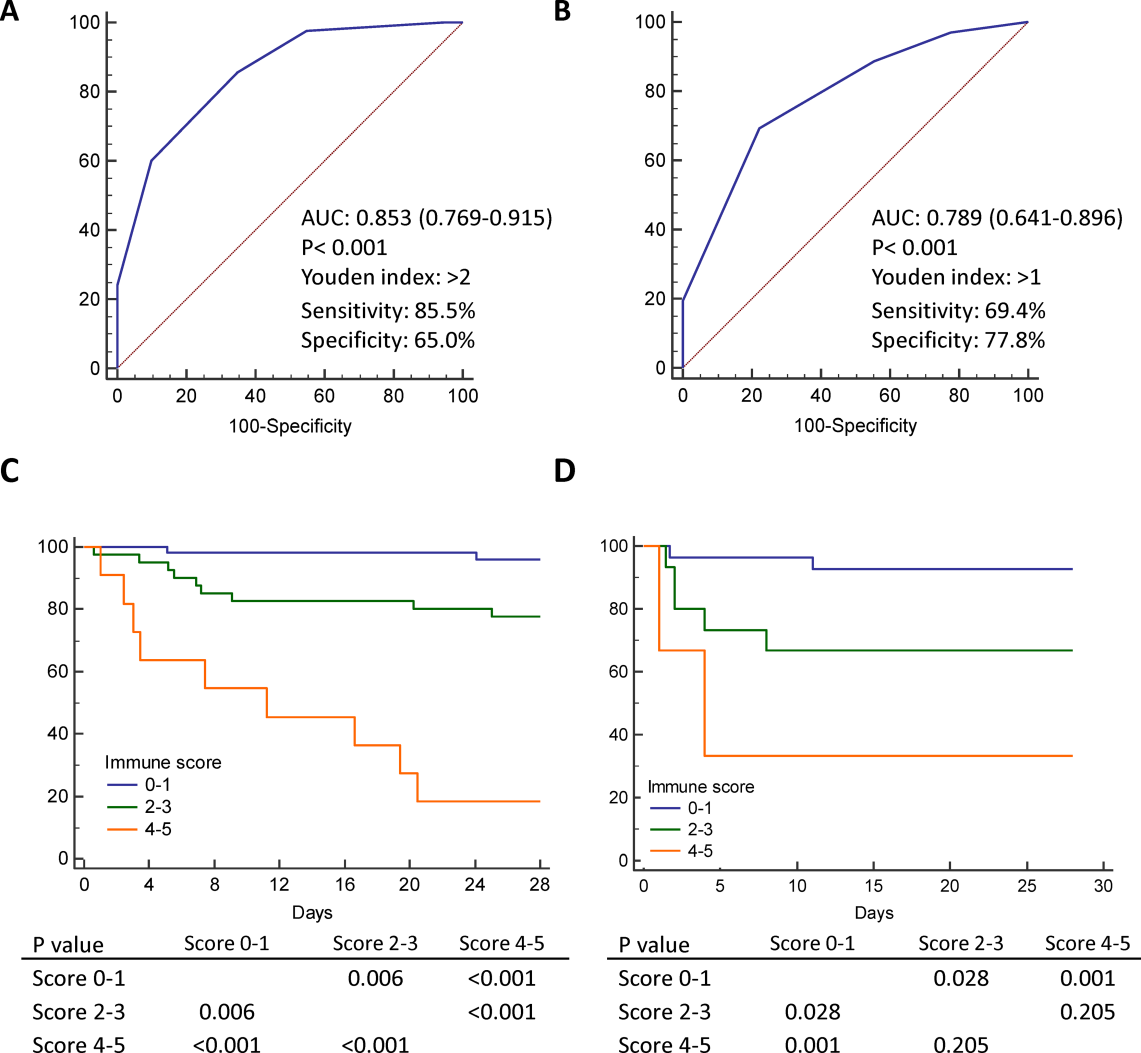
Receiver operating characteristic curve for discriminating between 28-day survivors and non-survivors with sepsis in the intensive care unit of the training (n = 103) (A) and validation (n = 45) (B) cohort. (C) Kaplan-Meier survival analyses of overall survival rates of patients with high, medium, and low immune dysfunction scores in the training cohort. (n = 103) (D) Overall survival rates of patients with high, medium, and low immune score in the validation cohort. (n = 45).

The score predicted 28-day mortality with an AUC of 0.853 in the training cohort (Fig 4A) that was validated in the validation cohort (Fig 4B). Patients were re-grouped into high (score 4–5), medium (score 2–3), and low (score 0–1) immune dysfunction levels. The Kaplan-Meier survival analyses of the low, medium, and high immune dysfunction levels are shown in Fig 4C, and the results were validated in the validation cohort (Fig 4D).

**Fig 5:**
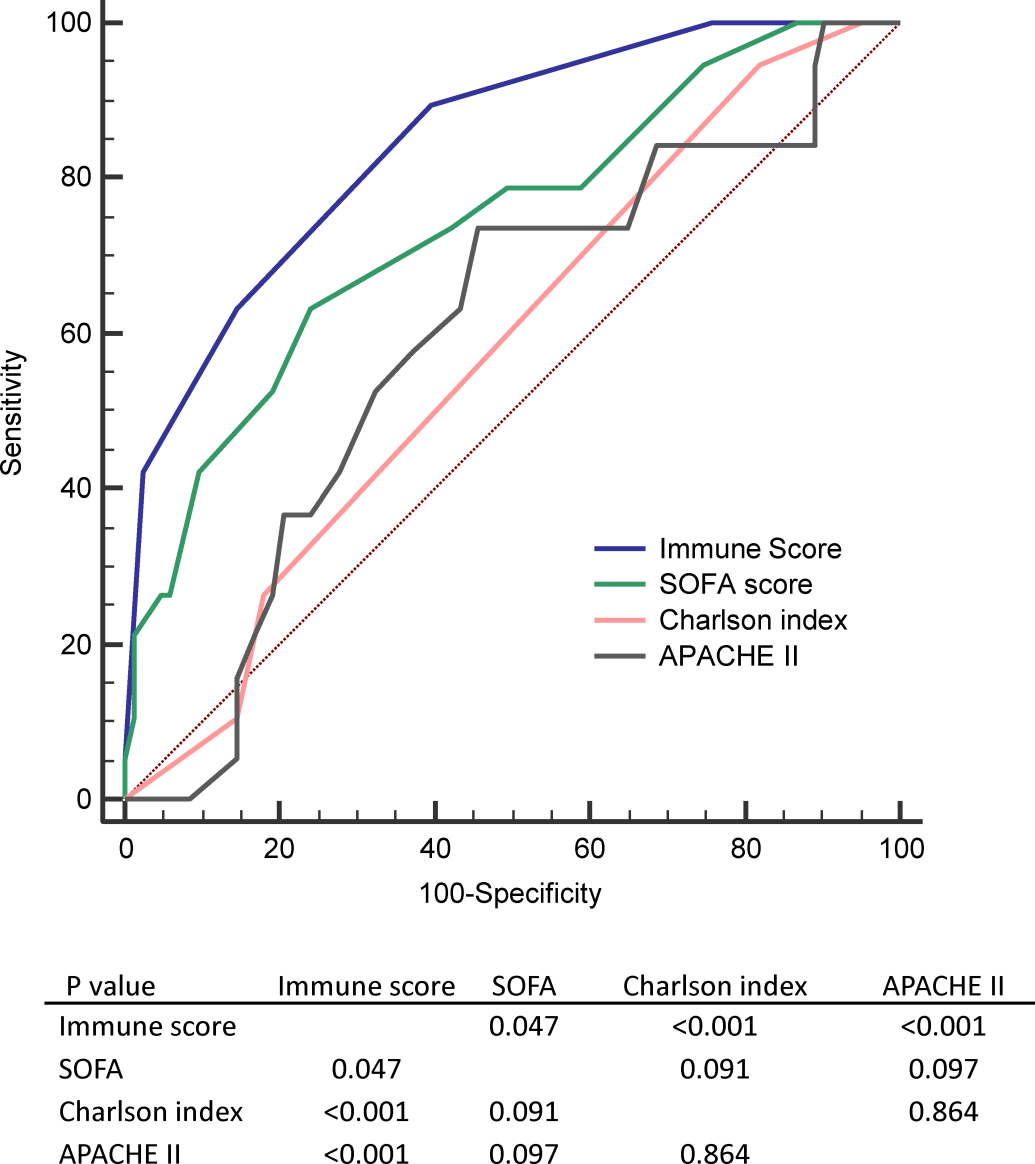
Receiver operating characteristic curve for comparing 28-day mortality prediction performance by using different scoring system in training cohort.

Performance measures of different scores including immune dysfunction score, SOFA score, Charlson index and APACHE II for 28-day mortality prediction were shown in Table 3 and Fig 5.

**Table 3:** Measure of performance predicting 28-day mortality by using different scoring systems(n = 102) ^a^.

	Sensitivity	Specificity	+LR	-LR	AUC
Immune score	65.0	85.54	4.50	0.41	0.846 (0.751–0.940)
SOFA	61.9	75.29	2.51	0.51	0.739 (0.611–0.867)
Charlson index	95.0	18.82	1.17	0.27	0.578 (0.460–0.734)
APACHE II	71.43	55.29	1.60	0.52	0.597 (0.446–0.710))

^a^ One patient had no baseline Charlson index.

### Stimulated immune response and immune dysfunction score

The stimulated immune response was evaluated using the cytokine elevation ratio, which was calculated by dividing the post–LPS-stimulated cytokine level by the pre–LPS-stimulated cytokine level. Of the 106 patients, 75 patients received stimulated immune response test.

**Fig 6:**
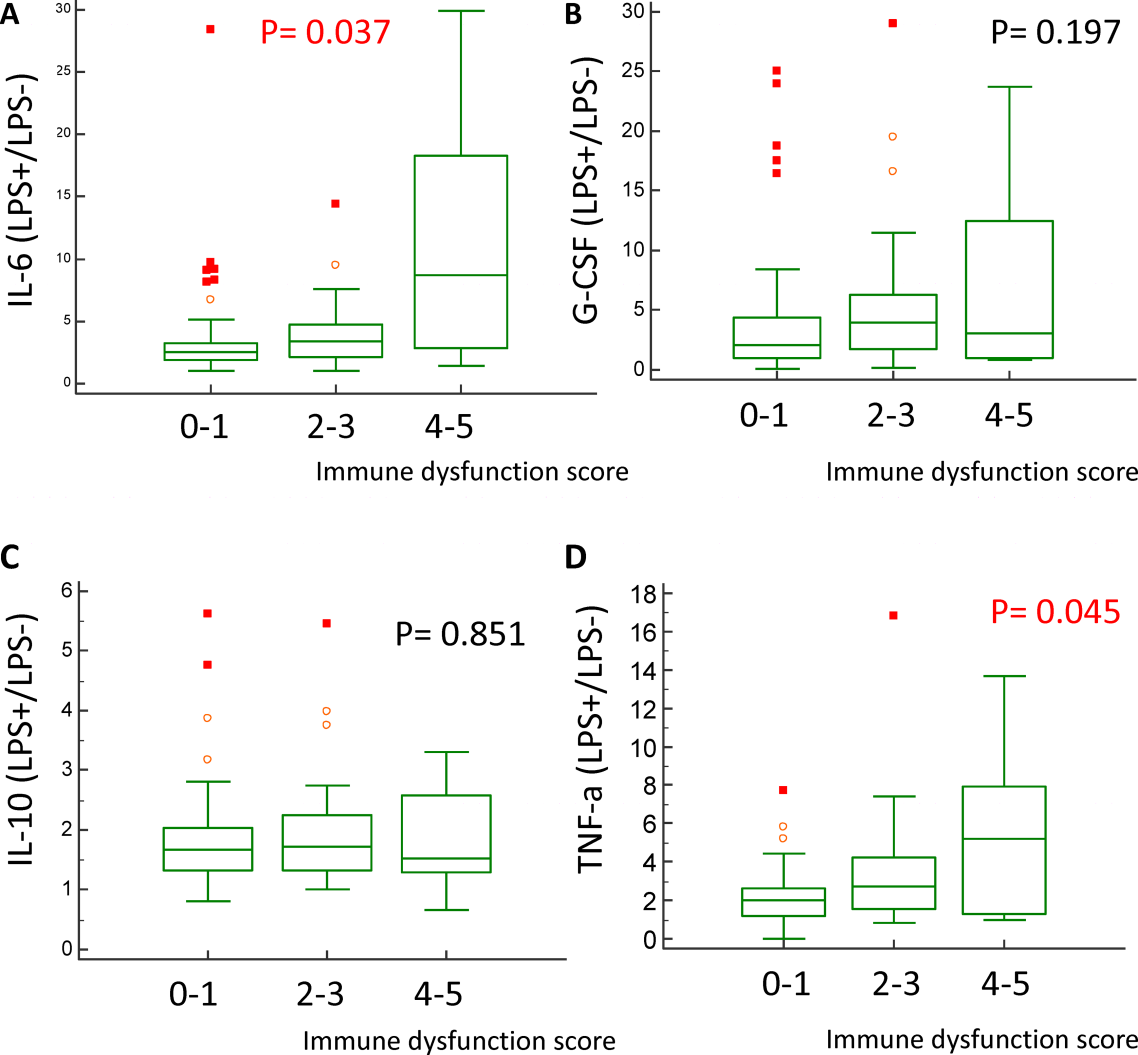
Association between post lipopolysaccharide stimulation immune response and immune dysfunction score 0–1, 2–3, 4–5 in training cohort using Kruskal-Wallis test (n = 75). Interleukin -6 (A), granulocyte-colony stimulating factor (B), interleukin -10 (C), and tumor necrosis factor-α (D).

The 28-day survivors had lower IL-6 and TNF-α elevation ratios after LPS stimulation than the non-survivors (Table 4). Patients with a higher immune dysfunction score also had higher IL-6 and TNF-α elevation ratios after LPS stimulation (Fig 6). Stimulated IL-10 and G-CSF ratios were not associated with 28-day mortality and immune dysfunction score.

**Table 4:** Ratio of immune response after LPS stimulation and 28-day mortality ^a^.

	All (N = 75) Median (IQR)	Non-survivors (n = 14) Median (IQR)	Survivors (n = 61) Median (IQR)	P value
G-CSF	2.06 (3.85)	3.70 (4.99)	2.01 (2.87)	0.230
IL-10	1.60 (0.78)	1.42 (1.17)	1.65 (0.73)	0.669
IL-6	2.68 (2.69)	4.60 (5.98)	2.62 (1.70)	0.017
TNF-α	2.09 (1.95)	2.85 (4.56)	2.05 (1.55)	0.027

^a^ Kruskal-Wallis test was used for assessing the association between post LPS stimulation immune response and immune dysfunction score.

G-CSF, granulocyte-colony stimulating factor; LPS, lipopolysaccharide; IL, interleukin; TNF-α, tumor necrosis factor-α

## Discussion

In our study, baseline immune parameters including decreased monocyte HLA-DR expression, higher plasma G-CSF level, higher plasma IL-10 level, and lower serum SeMo ratio were independent predictors of 28-day mortality in sepsis patients.

The immune response in patients with sepsis ranges from an exuberant pro-inflammatory cascade to a profoundly immunosuppressed phenotype [17]. The proper balance between the competing pro- and anti-inflammatory pathways determines the outcomes of patients with sepsis. The identification of immunoparalysis from immune storm is potentially important before widely application of immunomodulation therapies [18].

Decreased monocyte HLA-DR expression measured by flow cytometry is currently the best marker for monitoring immune alterations in critically septic patients [19]. The maladaptive immune dysfunction in patients with sepsis manifests across a range of cellular actions and functions that involve the innate and adaptive arms of the immune system. Defects have been noted in neutrophils and monocytes [20]. The effects of protracted sepsis on the innate immune system in macrophages include increased anti-inflammatory cytokine secretion, decreased anti-inflammatory cytokine secretion, decreased HLA-DR expression, and decreased pathogen killing. Higher monocyte counts contribute to improved outcomes after both PBMC and umbilical cord blood transplantations [21]. Monocytes may contribute to secondary injury after intracerebral hemorrhage [22]. In neutrophils, sepsis-related effects include immature neutrophil release, increased IL-10 secretion, and others [9]. Neutrophils are essential to the early control of invading pathogens. Neutrophil apoptosis is delayed during sepsis [23]. Infection control requires the efficient migration of neutrophils to the site of infection. The insufficient number of neutrophils recruited to the site of infection does not control the infection locally, contributing to the systemic spread of the pathogen [24].

Our results revealed that the SeMo ratio is an independent risk factor that can predict 28-day mortality. Monocytes from septic patients typically exhibit a diminished capacity to release pro-inflammatory cytokines such as TNF-α and IL-6, whereas the release of anti-inflammatory mediators such as IL-10 is neither impaired nor enhanced [9]. Although anti-inflammatory therapy (e.g., IL-10) makes sense during the initial hyperinflammatory phase, immune stimulation by the administration of monocyte-activating cytokines (interferon-γ, GM-CSF) may be useful during “immunoparalysis” [25]. GM-CSF therapy facilitates rapid recovery of immunoparalysis and prevents nosocomial infection [26, 27]. Clinical trials aimed at downregulating these mediators using antibodies against endotoxin have been uniformly disappointing. One of the reasons for such failure may be the lack of precise immunological parameters [28].

IL-10 overexpression on sepsis day 1 is suggestive of the overt anti-inflammation that is predictive of poor outcome. There are divergent subtypes within the heterogeneous syndrome of sepsis [17]. Most sepsis deaths in the ICU occur after a prolonged course, which is difficult to reproduce in animal models [29]. Sepsis-induced immune suppression leads to increased susceptibility to secondary infections with associated late mortality [30].

We also used immunologic monitoring by evaluating the monocyte cytokine production in patients with sepsis after endotoxin stimulation. Although these techniques involve in vitro analysis and are not widely available, this may help us understand immune dysfunction severity [31, 32]. In our series, patients with higher monocyte IL-6 and TNF-α production after re-exposure to LPS had higher 28-day mortality rates and immune dysfunction score. There was no association between IL-10, G-CSF expression and 28-day mortality rates as well as immune dysfunction score. As pro-inflammatory cytokines, serum and plasma TNF-α and IL-6 levels have been shown to increase significantly among patients with sepsis, particularly in those who are culture-positive [33, 34]. Some previous studies revealed immunosuppression consequent to monocyte desensitization to endotoxin that led to fatal outcomes [35]. However, these studies were mainly conducted in animals. Our study, unlike previous studies, revealed that patients with proinflammatory cytokine overproduction after LPS re-exposure had higher 28-day mortality rates [13, 36–38]. Inappropriately suppressed baseline levels of IL-6 and TNF-a in the unstimulated PBMCs may consequent to higher mortality rate.

The main strength of this study is its relatively large sample size with complete cytokine data and clinical correlation. However, there are several limitations worth noting. These limitations include selection bias, since all patients were recruited from a single medical center, and patient heterogeneity with regards to the sepsis source. Although patients with different sepsis sources are included in this analysis, the heterogeneity is reflective of the diverse phenotype of sepsis patients in the clinical setting. Furthermore, despite the differences in sepsis source among the patients studied, our immune dysfunction was able to be validated in a separate cohort, albeit relatively small. It is clear that a larger, more robust sample of patients will be required in the future for validation purposes to ensure the generalizability of the immune dysfunction scoring system to a broader patient population. In this study, patients with ICU waiting times longer than 24 hrs were excluded. Therefore, whether our results can be applied to patients with longer ICU waiting times has yet to be determined. Finally, the current inaccessibility to plasma cytokine levels and monocyte HLA-DR expression in the routine clinical setting prevents the widespread applicability of the immune dysfunction score. However, with further technological advancements and decreased costs in the future, it may be possible to incorporate these biomarkers into the clinical setting for risk stratification.

We are now also exploring the effects of dynamic immune status on other important outcomes in addition to 28-day mortality that are beyond the scope of this study.

## Conclusion

The immune dysfunction scoring system developed here incorporates plasma G-CSF level, IL-10 level, serum SeMo ratio, and monocyte HLA-DR expression and appears valid and reproducible for predicting 28-day mortality.

## Supporting information

S1 Table Demographics and clinical characteristics between the test and validation cohorts. (DOCX)

S1 File Raw data. (XLS)
