# Supplementary material for: Development and validation of immune dysfunction score to predict 28-day mortality of sepsis patients
Source: PLoS One. 2017 Oct 26;12(10):e0187088. doi: 10.1371/journal.pone.0187088 (PMC5658156; doi:10.1371/journal.pone.0187088)
Supplement: S1 Table — (DOCX) [file pone.0187088.s001.docx]

Demographics and clinical characteristics between the training and validation cohorts

| 28-day mortality | Test (n = 106) | Validation (n = 45) | P value |
| --- | --- | --- | --- |
| Age (years), mean (SD)a | 68.2 (15.7) | 65.2(13.1) | 0.262 |
| APACHE II score, mean (SD) | 26.8 (8.5) | 24.2(9.2) | 0.104 |
| Male, n (%)b | 61 (57) | 30 (66.7) | 0.295 |
| Body mass index, mean (SD) | 23.24 (5.1) | 23.0(4.7) | 0.807 |
| Charlson index, mean (SD) | 2.42 (1.5) | 2.7(2.1) | 0.350 |
| Cardiovascular disease, n (%) | 32 (30) | 13 (28.9) | 0.873 |
| Hypertension, n (%) | 64 (60) | 23 (51.1) | 0.292 |
| COPD, n (%) | 18 (17) | 4 (8.9) | 0.312 |
| Asthma, n (%) | 5 (5) | 1 (2.2) | 0.670 |
| Pulmonary tuberculosis, n (%) | 4 (4) | 2(4.4) | 1.000 |
| Cancer, n (%) | 15 (14) | 14(31.1) | 0.016 |
| Diabetes mellitus, n (%) | 55 (52) | 20 (44.4) | 0.403 |
| Stroke, n (%) | 24 (23) | 8 (17.8) | 0.504 |
| Chronic kidney disease, n (%) | 28 (26) | 6 (13.3) | 0.078 |
| SOFA score, mean (SD) | 9.45 (3.6) | 9.6(3.6) | 0.767 |
| WBC, 1000/µL, mean (SD) | 16.27 (8.5) | 17.9(10.2) | 0.304 |
| SeMo ratio, mean (SD) | 30.97 (50.3) | 30.4(29.9) | 0.945 |
| C-reactive protein, mg/L, mean (SD) | 171.79 (128.0) | 198.0(148.1) | 0.314 |
| Procalcitonin, ng/mL, mean (SD) | 23.93 (49.7) | 34.6(56.7) | 0.353 |
| G-CSF pg/mL, mean (SD) | 63.09 (104.8) | 1241.2(3223.4) | 0.376 |
| IL-10, pg/mL, mean (SD) | 15.25 (58.05) | 112.2(330.9) | 0.965 |
| IL-6, pg/mL, mean (SD) | 43.31 (84.3) | 652.2(2401.8) | 0.186 |
| TNF-α pg/mL, mean (SD) | 31.15 (36.5) | 88.1(192.7) | 0.082 |
| HLA-DR expression %, mean (SD) | 90.30 (18.4) | 89.7(13.9) | 0.102 |
